# Evaluation of the Reversibility of Cadmium-Induced Testicular Toxicity Following Recovery Alone or with Zinc Supplementation

**DOI:** 10.3390/ijerph22030454

**Published:** 2025-03-20

**Authors:** Jihane Ait Benbella, Samy Housbane, Youness Kadil, Fatimaezzahra Kabbali, Ikram Ghicha, Hasnaa Bazhar, Fatiha Bousselham, Afaf Banid, Othmane Hammani, Noureddine Louanjli, Mehdi Karkouri, Abderrahmane Mellouki, Houda Filali, Rachid Aboutaieb

**Affiliations:** 1 Laboratory of Sexual and Reproductive Health, Faculty of Medicine and Pharmacy, Hassan II University Casablanca, Casablanca 20360, Morocco; 2 Department of Medical Informatics, Faculty of Medicine and Pharmacy, Hassan II University Casablanca, Casablanca 20360, Morocco; 3 Laboratory of Clinical Pharmacology and Toxicology, Faculty of Medicine and Pharmacy, Hassan II University Casablanca, Casablanca 20360, Morocco; 4 National Center for Scientific and Technical Research (CNRST), Rabat 10102, Morocco; 5 Labomac IVF Center and Clinical Laboratory Medicine, Casablanca 20120, Morocco; 6 Department of Pathology, CHU Ibn Rochd, Casablanca 20100, Morocco; 7 Department of Urology, CHU Ibn Rochd, Casablanca 20100, Morocco

**Keywords:** cadmium, testicular toxicity, recovery, zinc

## Abstract

Cadmium (Cd) is a toxic heavy metal that disrupts spermatogenesis and steroidogenesis due to its long half-life. This study evaluated the impact of recovery alone or with zinc (Zn) supplementation on Cd-induced testicular toxicity. A total of 42 pubertal male Wistar rats were divided into seven groups of six rats each. The control group (1) received NaCl (0.9%). Groups 2, 3, and 4 were treated with Cd 10 μg/kg/d by intraperitoneal injection for 1, 2, and 3 months respectively. Group 5 received Cd for 3 months with a recovery period of 1 month; Group 6 was exposed to Cd for 3 months, followed by a 1-month recovery period combined with Zn supplementation. (0.5 mg/kg/d). The last group was treated with zinc at a dose of 0.5 mg/kg/day for one month. The results showed decreased body weight, testicular and epididymal weight, testicular dimensions, and sperm parameters, along with Cd accumulation in the testes. Cd caused testicular damage and reduced serum testosterone levels, with more pronounced effects in the 3-month treatment group. Recovery alone did not significantly reverse Cd’s toxic effects, whereas Zn supplementation mitigated most of the damage. Recovery combined with Zn supplementation was more effective in correcting Cd-induced testicular toxicity than recovery alone.

## 1. Introduction

Infertility is a significant clinical concern and a global public health issue. It is defined as the inability of a couple to conceive after 12 months or more of regular, unprotected sexual intercourse, affecting approximately 15% of couples of reproductive age. Worldwide, an estimated 70 million couples experience hypofertility or infertility [1].

Male factors account for 40 to 50% of infertility cases in couples. Diagnosing male infertility requires an understanding of its pathophysiology and underlying causes.

The causes of male infertility are diverse, including genetic mutations, mechanical issues (e.g., varicocele and retrograde ejaculation), hormonal imbalances (e.g., hypogonadism), and poor sperm quality, which is implicated in 90% of male infertility cases [2]. Multiple factors contribute to decreased sperm quality, including environmental and behavioral toxicants, such as alcohol, smoking, drug use, air pollution, excessive heat, stress, chemicals, and poor dietary habits, all of which are increasingly studied for their impact on male fertility [3].

Cadmium (Cd) is a highly toxic transition metal with unknown biological function. Its presence in the environment, primarily due to zinc and lead mining and metallurgical activities [4], as well as other industrial sources, poses a major threat to human and animal health [5]. Cd exposure primarily occurs through environmental pathways, including air, water, and food, due to soil and crop contamination from fertilizers, pesticides, and wastewater [6]. Additionally, occupational exposure to Cd occurs in industries such as battery manufacturing, pigment production, plastics, and metal alloy processing [7]. Smoking is also a significant source of Cd exposure, as tobacco leaves naturally accumulate Cd, which is inhaled during smoking [8].

Once absorbed, Cd is poorly excreted and accumulates in the body, mainly in the liver and kidneys, where it can represent 50–75% of the total body burden [9]. Its long biological half-life, estimated between 20 and 40 years, is due to inefficient excretion mechanisms [10]. Intracellularly, Cd is primarily complexed with metallothionein (MT), a low-molecular-weight protein involved in metal homeostasis and detoxification [11]. However, this interaction also promotes renal Cd accumulation, contributing to its chronic toxicity.

The testes are a major target for Cd accumulation, leading to deleterious effects on reproductive function [12]. Cd enters the seminiferous tubules by altering the endothelial cells of testicular blood vessels, thereby disrupting the blood-testis barrier (BTB) and facilitating metal diffusion into germ cells [13]. This accumulation leads to severe dysfunction in Sertoli and Leydig cells, impairing the structural support of germ cells and disrupting testosterone production [14].

One of the primary mechanisms of Cd toxicity in the testes is oxidative stress. Cd generates reactive oxygen species (ROS), disrupting the balance between free radical production and antioxidant defenses [15], resulting in cellular damage. This oxidative toxicity impairs spermatogenesis, reduces sperm count and quality, decreases motility, and increases morphological abnormalities [16]. Cd also disrupts mitochondrial function, exacerbating ROS production, inducing lipid peroxidation of cell membranes, causing oxidative DNA damage, and activating apoptotic pathways leading to programmed cell death [17]. Additionally, some studies suggest that Cd may act as an endocrine disruptor by interacting with androgen and estrogen receptors [18], further contributing to hormonal imbalances that exacerbate reproductive dysfunction.

In contrast to cadmium, zinc is an essential trace element involved in numerous biological processes throughout the body. It plays a critical role in male reproductive function, including sperm maturation in the epididymis, testosterone production, and testicular development [19]. Zinc deficiency has been linked to reduced sperm fertilization capacity and hypogonadism in men [20]. Furthermore, zinc deficiency can exacerbate Cd toxicity by promoting its accumulation in the testes [21].

Cadmium exhibits chemical similarities to essential divalent metals, such as iron (Fe^2^⁺) and zinc (Zn^2^⁺), the latter being in the same group of the periodic table [22]. Due to this similarity, Cd can interfere with biological processes by substituting for Zn^2^⁺ or mimicking its action, disrupting the activity of zinc-dependent enzymes [23]. This improper substitution impairs various metabolic pathways, including protein synthesis, oxidative stress regulation, and cellular integrity maintenance. Additionally, Cd’s high affinity for certain metal transporters facilitates its absorption and accumulation in tissues, exacerbating its toxic effects.

This study aims to examine the toxic effects of Cd on rat testes following chronic exposure to low doses and to evaluate the reversibility of these effects through recovery alone or in combination with Zn supplementation.

## 2. Materials and Methods

### 2.1. Chemicals

Cadmium chloride (CdCl_2_) and zinc chloride (ZnCl_2_.H_2_O), with a purity of 98% were obtained from Scomalab Sarl (Oxford, Oxford, UK).

### 2.2. Animals

Forty-two adult male Wistar rats (3–4 months old) were housed in individually ventilated plastic cages under controlled environmental conditions: a temperature range of 20–24 °C, 55 ± 10% humidity, and a 12/12-h light/dark cycle. They were provided with a commercially balanced diet and had ad libitum access to tap water throughout the experimental period. The study was approved by the Biomedical Research Ethics Committee of the Faculty of Medicine and Pharmacy, Casablanca, Morocco (No. 05/2023).

### 2.3. Experimental Design

Forty-two male Wistar rats were randomly divided into seven groups (six rats per group). Group 1 remained untreated and served as the control, receiving only physiological saline (0.9% NaCl). Five groups received an identical dose of cadmium (10 μg/kg b.w) for three different time durations as follows: Group 2 received CdCl_2_ for one month (Cd1), Group 3 for two months (Cd2), and Group 4 for three months (Cd3). Group 5 was exposed to CdCl_2_ for three months followed by a one-month recovery period (Cd3recov), and Group 6 underwent the same exposure but received a zinc supplementation of 0.5 mg/kg b.w of ZnCl_2_ during the recovery phase (Cd3recov + Zn). The last group (Group 7) served as a positive control and received 0.5 mg/kg b.w of ZnCl2 (Zn) [24,25]. 0.9% NaCl, CdCl_2_, and ZnCl_2_ were administered intraperitoneally.

These groups were used in two studies: the first aimed to evaluate Cd toxicity in the testes at three time points (1, 2, and 3 months) to assess the progression of Cd-induced alterations over time. For this purpose, the control group, along with the Cd1, Cd2, and Cd3 groups were used.

The second study evaluated the reversibility of Cd toxicity in the testes by assessing the effects of a one-month recovery period, with or without zinc supplementation, after three months of Cd exposure. For this study, the control group, Cd3, Cd3recov, Cd3recov + Zn, and Zn groups were used.

### 2.4. Determination of Sperm Parameters

The caudal epididymis of each rat was separated from the testis, placed on a paper towel to remove excess fluid, weighed, then lacerated and incubated in 2 mL of preheated saline at 37 °C for 5 min. The resulting suspension was used for further analysis [26].

#### 2.4.1. Epididymal Sperm Count

The semen suspension was diluted at a ratio of 1:9 or 1:19 depending on the initial concentration. A 10 μL aliquot of the diluted semen was placed in each chamber of a modified Neubauer hemocytometer (Marienfeld, Lauda-Königshofen, Germany). The sperm count was performed under an optical microscope (×40) in the five corners and the center of the chamber. The sperm count was expressed as ×10^6^ mL⁻^1^ [26].

#### 2.4.2. Sperm Mobility

A 50 μL aliquot of the suspension was placed on a glass slide. One hundred spermatozoa were observed in 10 different fields under an optical microscope (×40) over 2–4 min. Sperm were classified as motile (A + B) or non-motile, and motility was expressed as a percentage [26].

#### 2.4.3. Sperm Viability

Approximately 50 μL of the suspension was mixed with a drop of 1% eosin solution. After 15 s, two drops of 10% nigrosine solution (Biognost, Zagreb, Croatia) were added. A drop of this mixture was transferred onto a glass slide, spread into a thin smear, and air-dried. Viable sperm appeared white, while dead sperm were stained pink. One hundred spermatozoa were counted under a microscope (×40), and viability was expressed as a percentage [24].

#### 2.4.4. Sperm Morphology

A drop of semen was spread onto a slide and air-dried or oven-dried at 37 °C. The smears were then fixed and stained using the Shorr staining method (Biognost, Zagreb, Croatia). Two hundred sperm were analyzed, and morphology was expressed as a percentage [27].

### 2.5. Measurement of Cadmium and Zinc Levels in the Testicles

One gram of fresh testicular tissue was weighed, followed by the addition of 10 mL of concentrated nitric acid (HNO_3_, 65%). The mixture was left to react overnight under a fume hood. The next day, the solution was heated on a hot plate at 80 °C until it became colorless and transparent. Deionized water was then added to reach a total volume of 10 mL. Samples were analyzed for Zn and Cd using Inductively Coupled Plasma Atomic Emission Spectroscopy (ICP-AES) [28]. The analysis was performed using a Horiba Scientific Jobin Yvon Ultima Expert instrument (Longjumeau, France), equipped with a peristaltic pump for sample introduction. The measured wavelengths were 213.857 nm for Zn and 214.441 nm for Cd. The operational parameters included an RF power of 1000 W, a plasma gas flow rate of 15 L/min, and a nebulizer gas flow rate of 0.7 L/min of argon. Element concentrations were calibrated using a 9-point calibration curve ranging from 0 to 5 mg/L with certified standard solutions. Cd and Zn concentrations were expressed in μg/g and mg/g of testicular tissue, respectively.

### 2.6. Assessment of Testosterone

After euthanasia under anesthesia, blood was collected, centrifuged (2500× *g*, 10 min), and the serum was stored at −80 °C until analysis. Testosterone levels, expressed in ng/mL, were determined using the Elecsys Testosterone II kit (Basel, Switzerland).

### 2.7. Histopathological Analysis

The left testis was fixed in 10% buffered formalin for 24 h, and then subjected to a dehydration process through an ascending ethanol series. After xylene treatment, the samples were embedded in paraffin blocks, sectioned into 5 μm thick slices, and stained with Harris hematoxylin and eosin for microscopic examination [29].

### 2.8. Statistical Analysis

Data are presented as mean ± SD. The normality assumption for ANOVA was assessed by analyzing the model residuals using the Shapiro–Wilk test. All ANOVA tests showed a Shapiro–Wilk normality *p*-value > 0.05. The homogeneity of variance assumption was checked using Levene’s test, with all ANOVA tests yielding a Levene’s test *p*-value > 0.05.

Statistical analyses were performed using one-way analysis of variance (ANOVA). A *p*-value of <0.05 was considered statistically significant, while a *p*-value of <0.01 indicated high statistical significance. All statistical procedures were conducted using the R Statistical Package.

## 3. Results

No clinical signs of toxicity were observed during the experimental period, and no rats were excluded from the study. Additionally, no mortality was recorded in either the treated or untreated groups.

### 3.1. Toxic Effect of Cadmium on Rat Testes at Three Time Points

#### 3.1.1. Body Weight, Testicular and Epididymal Weights, and Testicular Dimensions

As shown in Table 1, body weight significantly decreased in the treated groups compared to the control group. This decrease was more pronounced in the groups treated for 2 and 3 months (*p* < 0.01) compared to the group treated for 1 month (*p* < 0.05).

The weight of the testes, as well as their dimensions (length, width, and depth), and the weight of the epididymis significantly decreased (*p* < 0.05) in the cadmium-treated groups. Specifically, the decrease in testicular weight and width was significant in the groups treated for 2 and 3 months (*p* < 0.01), while the length of the testes was significantly reduced only in the group treated with Cd for 3 months.

#### 3.1.2. Analyses of Sperm Parameters

Compared with the control group, sperm count, mobility, viability. and morphology significantly decreased (*p* < 0.01) in the Cd-treated groups. The group treated with Cd for 3 months showed a more pronounced decrease compared to the groups treated for 1 and 2 months (Table 2).

#### 3.1.3. Testosterone Assay

Testosterone levels were significantly lower in the group treated with Cd for 2 and 3 months (*p* < 0.05 and *p* < 0.01, respectively) compared to the control group. A slight decrease in testosterone was observed in the group treated for 1 month (Table 3).

#### 3.1.4. Cadmium and Zinc Levels in the Testes

Compared to the control group, Cd concentration significantly increased in all treated groups (*p* < 0.01). Conversely, zinc levels significantly decreased in the Cd-treated groups, as shown in Table 4.

#### 3.1.5. Testicular Histology

**Figure 1:**
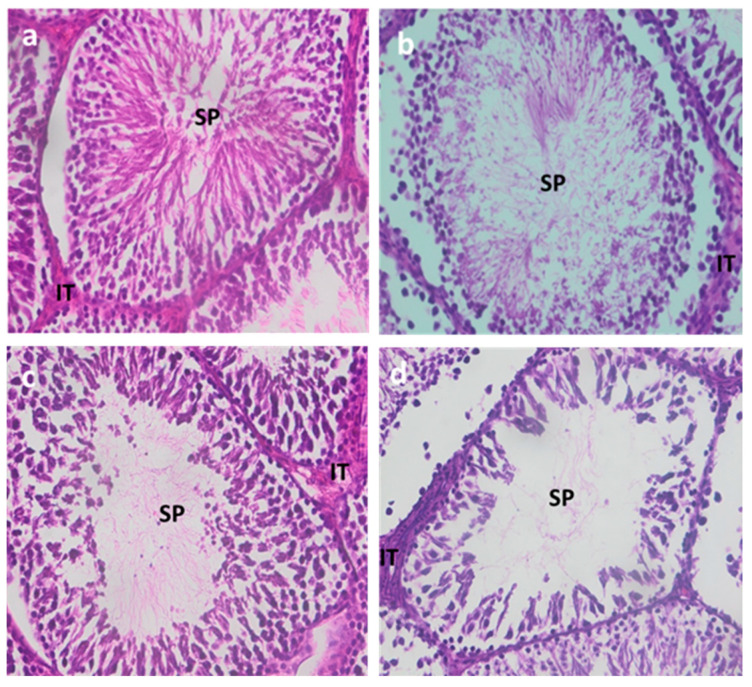
Histological sections of the testes of the control (**a**) and Cd rats treated for one month (**b**), two months (**c**), and three months (**d**). IT: interstitial tissue, SP: spermatozoa.

Histological sections of the testes from the control group exhibited a generally preserved architecture, with seminiferous tubules showing normal morphology, well-organized cell layers, and a normal sperm count (Figure 1a).

Rats treated with Cd for varying durations showed distinct histopathological changes.

After 1 month of Cd exposure (Figure 1b), seminiferous tubules displayed a very minimal reduction in the number of cell layers, and various cells and spermatozoa were present but very slightly reduced, along with a slight disorganization of the cell layers.

After 2 months, damage becomes more pronounced, with increased disorganization of cell layers and a further reduction in spermatozoa (Figure 1c).

After 3 months, a moderate to severe reduction in spermatozoa in the seminiferous tubules was observed, along with moderate disorganization of cell layers and a reduction in their number (Figure 1d).

### 3.2. Study of the Reversibility of Cadmium Toxicity

#### 3.2.1. Body Weight, Testicular and Epididymal Weights, and Testicular Dimensions

No significant changes were observed in the group treated with Zn compared to the control group (Table 5).

After 1 month of recovery following 3 months of Cd exposure, there was no significant increase in body weight. However, Zn supplementation during the recovery period led to a significant improvement compared to the group treated with Cd for 3 months (*p* < 0.05).

There was significant improvement in testicular weight, length, or epididymal weight during recovery, with or without zinc supplementation (Table 5). However, the width and depth of the testes significantly increased in the group treated with Zn during recovery (*p* < 0.01), while recovery alone led to a significant increase only in testicular width (*p* < 0.01).

#### 3.2.2. Analyses of Sperm Parameters

**Figure 2:**
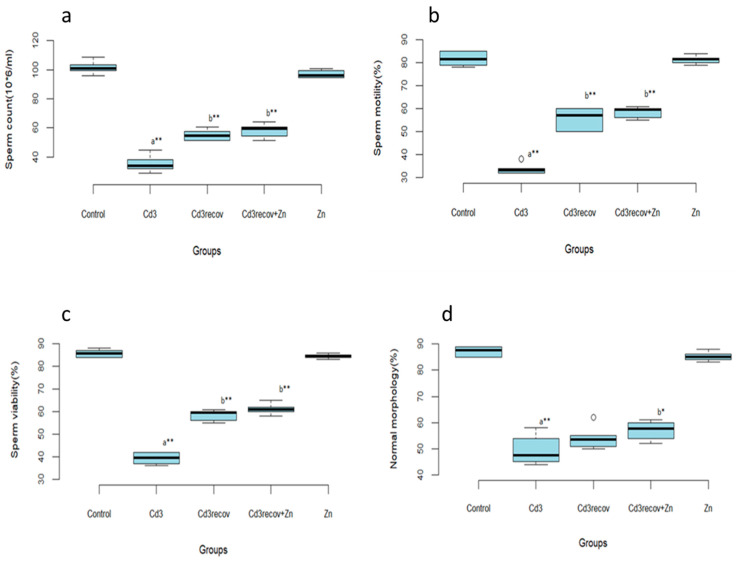
Impact of recovery alone or with zinc supplementation on sperm parameters after Cd for 3 months of Cd treatment. Sperm count (**a**); sperm motility (**b**); sperm viability (**c**); normal morphology (**d**); means ± SD of six animals in each group, statistically significant differences are indicated as follows: ^a^ vs. control, ^b^ vs. Cd3, * *p* < 0.05, ** *p* < 0.01.

After 1 month of recovery, sperm count, motility, and viability significantly improved (*p* < 0.01) compared to the group treated with Cd for 3 months. However, sperm morphology did not show a significant improvement. Zn supplementation during the recovery period led to significant improvements in all sperm parameters compared to the group treated with Cd for 3 months (Cd3) (Figure 2).

#### 3.2.3. Testosterone Assay

**Figure 3:**
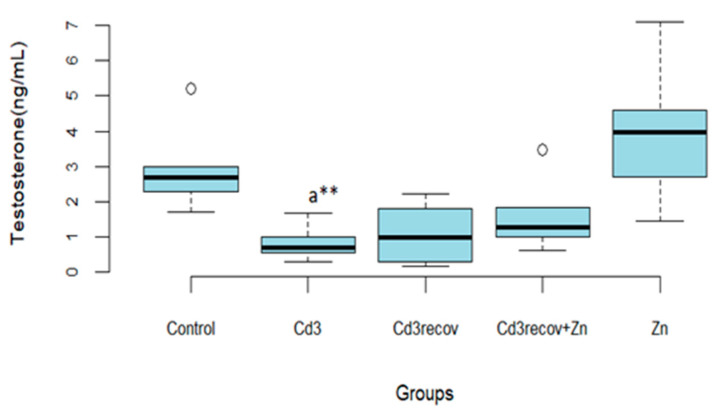
Influence on recovery alone or in combination with Zn on plasma testosterone levels in the group treated with Cd for 3 months. Means ± SD of six animals in each group, statistically significant differences are indicated as follows: ^a^ vs. control, ** *p* < 0.01.

Recovery for 1 month alone or with zinc supplementation only resulted in a slight improvement in testosterone levels compared to the group treated with Cd for 3 months (Figure 3).

Zn administration had no significant effect on testosterone production in rats compared to the control group.

#### 3.2.4. Cadmium and Zinc Levels in the Testes

**Figure 4:**
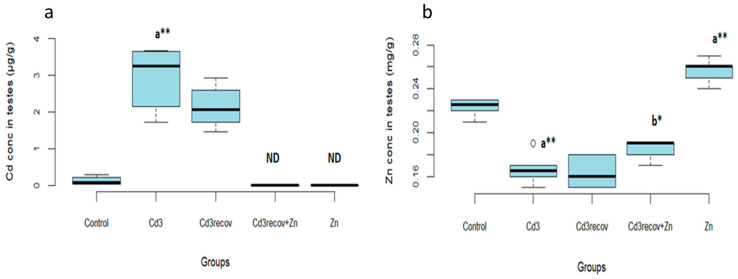
Concentration of Cd (**a**) and Zn (**b**) in the testes of rats. Means ± SD of six animals in each group, statistically significant differences are indicated as follows: ^a^ vs. control, ^b^ vs. Cd3, * *p* < 0.05, ** *p* < 0.01, ND: non-detectable.

One month of recovery without zinc supplementation did not significantly reduce Cd concentration in the testes compared to the group treated with Cd for 3 months (Figure 4a).

However, zinc supplementation during the recovery period significantly reduced Cd concentration in the testes (*p* < 0.05). In addition, Zn levels in the testes significantly increased in the zinc-treated group (*p* < 0.01) compared to the control group (Figure 4b).

#### 3.2.5. Testicular Histology

**Figure 5:**
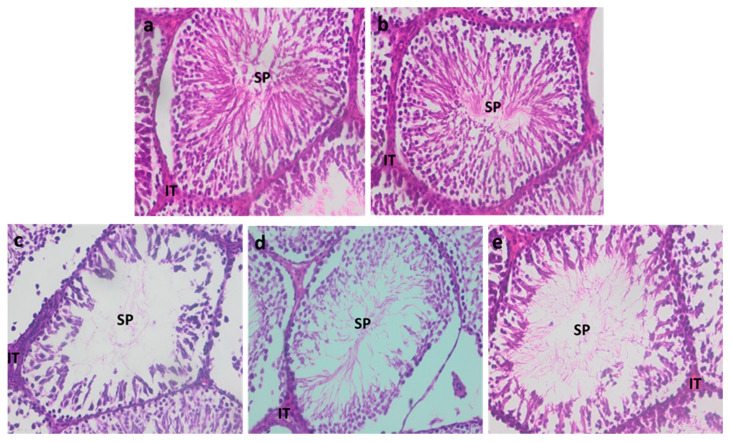
Histological sections of the testes from control rats: (**a**) rats treated with Zn alone (**b**) treated with Cd for three months (**c**) rats that recovered for 1 month, (**d**) rats that recovered for 1 month with Zn supplementation, and (**e**) abbreviations on the image: IT: interstitial tissue, SP: spermatozoa.

Histological sections of the testes from the control group and the zinc-treated group showed a well-preserved architecture, with normal seminiferous tubules, well-organized cell layers, and normal sperm count (Figure 5a,b).

Recovery, either alone (Figure 5d) or in combination with zinc (Figure 5e), promoted an increase in sperm count, the number of cell layers, and improvement in their organization, compared to the Cd-treated group (Cd3) (Figure 5c).

## 4. Discussion

The primary objective of this study was to assess the impact of chronic cadmium (Cd) exposure on testicular health in male Wistar rats, with a particular focus on the reversibility of these effects after cessation of exposure, either alone or in combination with zinc (Zn) supplementation. What sets this study apart from previous research is that we specifically investigated recovery following chronic Cd exposure, as well as the effect of zinc supplementation administered after the cessation of Cd exposure, rather than during the exposure itself. This innovative experimental design allowed us to explore the efficacy of zinc as a therapeutic intervention post-exposure—an aspect that remains underexplored in the existing literature.

Cadmium poses a significant threat to human health due to continuous and involuntary exposure, primarily through environmental sources, such as contaminated drinking water and food. Additional exposure can occur in occupational settings where Cd is used in various industrial processes, as well as through behaviors such as smoking. The long biological half-life of Cd in the human body, ranging from 20 to 40 years [10], contributes to its bioaccumulation and high toxicity, even at low doses.

We observed a significant decrease in the body weight of rats exposed to Cd, along with a reduction in the weight of the testes and epididymis, as well as a decrease in testicular dimensions. These findings are consistent with previous studies [24,26,30]. However, our study assessed these parameters at three different time points, with the most pronounced effects observed in the group exposed to Cd for three months. It has been demonstrated that Cd disrupts digestive enzymes and impairs protein absorption [31]. This reduction in the intake of essential nutrients could partially explain the observed decrease in body weight. The decline in testicular weight and dimensions may be attributed to a reduction in germ cell density [24], increased apoptosis of testicular tissue [20,32], or impaired testosterone biosynthesis [29].

The testes are highly sensitive to cadmium (Cd) compared to other organs in the body. Cadmium has been shown to be both spermotoxic and an endocrine disruptor [10]. In rats, body weight serves as an indicator of overall health, while testicular weight and dimensions are particularly crucial in toxicity studies [26,33].

Furthermore, Cd exposure led to significant alterations in sperm parameters, with the most pronounced effects observed in the group exposed to Cd for three months. The key mechanism underlying sperm parameter decline is the induction of oxidative stress. The sperm plasma membrane, rich in polyunsaturated fatty acids, is particularly vulnerable to oxidative damage [26,34]. Cd has been implicated in the excessive production of reactive oxygen species (ROS) through the Fenton reaction by mimicking iron and copper in certain enzymatic processes [17]. This phenomenon leads to lipid peroxidation and disruption of sperm membrane integrity, thereby explaining the reduction in sperm motility and viability. Additionally, Cd can decrease glutathione (GSH) concentration by binding to thiol (-SH) groups, thereby impairing the antioxidant activity of various enzymes [35]. Unlike previous studies that primarily focused on Cd exposure or preventive measures during exposure, our study specifically investigated the recovery phase following chronic Cd exposure. While prior research has demonstrated that partial recovery of sperm parameters (count, motility, viability, and morphology) is possible after one month of Cd exposure cessation [30], our study went further by assessing this recovery after prolonged Cd exposure. Our findings indicate that, even after chronic exposure, partial recovery of sperm parameters can be achieved within one month of cessation. This recovery suggests that certain toxic effects may be mitigated, yet it remains incomplete, particularly concerning hormonal function.

Testosterone is primarily synthesized in Leydig cells and subsequently utilized by Sertoli cells to regulate spermatogenesis. It serves as a survival factor for germ cells, protecting them from apoptosis, and its depletion leads to impaired sperm parameters, reduced testicular weight, and histological alterations [36].

Our results confirm that cadmium acts as a major endocrine disruptor by reducing testosterone levels [37]. The observed decrease in testosterone in our study aligns with previous reports of similar effects [26,30,38]. However, what sets our study apart is the analysis of the effects of exposure cessation and zinc supplementation after chronic Cd exposure, a topic that has not been sufficiently explored in previous studies. Interestingly, although testosterone levels showed partial improvement following zinc supplementation, they did not return to control levels. This suggests that while partial reversibility of hormonal functions is possible, complete recovery may require a longer period or prolonged supplementation. This decline is likely linked to dysfunction in Leydig cells, which are responsible for testosterone production, as evidenced by reduced testosterone levels [39,40]. Following Cd exposure, the metal can accumulate within the hypothalamic-pituitary-testicular (HPT) axis [41], affecting all three regulatory levels. Additionally, oxidative stress and lipid peroxidation have been identified as key mechanisms by which Cd exerts toxicity on the pituitary gland [42], affecting all three regulatory levels. Additionally, oxidative stress and lipid peroxidation have been identified as key mechanisms by which Cd exerts toxicity on the pituitary gland [43]. Another critical factor to consider is the effect of Cd on androgen receptors. It has been suggested that Cd may interact with these receptors, inhibiting their activation and thereby reducing the biological response to testosterone [44].

We observed a significant accumulation of Cd in the testes of rats after three months of exposure. Cd exposure also reduced Zn concentration in the testes. The elimination of cadmium, even one month after exposure cessation, appears to be a slow process, which explains the persistence of certain toxic effects. Studies have demonstrated that Cd can cause renal dysfunction due to its long biological half-life, thereby slowing the excretion of the metal from the body [45]. The reduction in Zn levels is attributed to its sequestration by hepatic and renal metallothioneins (MTs) induced by Cd [46].

Zinc supplementation, administered exclusively after Cd exposure cessation, demonstrated a significant beneficial effect by reducing Cd accumulation in the testes and promoting the recovery of both sperm and testicular parameters. This observation highlights a unique aspect of our study—the introduction of zinc supplementation only after Cd exposure had ended. These findings emphasize the therapeutic potential of zinc in protecting and repairing testicular tissues following chronic Cd exposure, demonstrating that post-exposure intervention can yield beneficial effects on testicular function.

It is well documented that zinc plays a crucial role as an antioxidant and enzymatic cofactor, contributing to the reduction of ROS indirectly generated by Cd toxicity [47]. Zinc functions as a cofactor in the active sites of enzymes that neutralize free radicals and can induce the synthesis of metallothioneins, which help reduce hydroxyl radicals (OH) and neutralize or eliminate ROS produced by Cd toxicity [47,48]. These mechanisms may explain zinc’s role in protecting cells against oxidative stress and preventing Cd accumulation in the testes [32]. Zn decreases Cd concentrations in the testes by inducing the synthesis of hepatic metallothioneins, which sequester Cd in liver tissues. In cases of Zn deficiency, Cd is released from MTs and accumulates in testicular tissues [49,50].

Finally, although zinc supplementation has demonstrated positive effects in regenerating testicular tissues, several questions remain regarding the precise mechanisms underlying this protection. Further exploration of the interaction between zinc and cadmium is warranted, particularly through the analysis of metallothioneins and oxidative stress markers in testicular tissues. Additionally, extending the recovery period would be valuable in determining whether a complete reversal of Cd’s toxic effects is achievable, potentially paving the way for new therapeutic strategies following Cd exposure.

## 5. Conclusions

In summary, the current study suggests that chronic exposure to cadmium, even at low doses, induces spermiotoxicity, steroidotoxicity, and testicular damage.

Recovery or cessation of cadmium treatment did not completely reverse the negative effects of cadmium exposure, except for improvements in sperm parameters and a reduction in cadmium concentration in the testes. However, zinc supplementation during the recovery period successfully reversed the toxic effects of cadmium on most of the parameters evaluated in this study.

## Figures and Tables

**Table 1:** Effect of Cd exposure for 1, 2, and 3 months on body weight, testicular and epididymal weights, and testicular dimensions.

		Control	Cd1	Cd2	Cd3
Final body weight		307.0 ± 12.36	264.0 ± 43.76 *	253.3 ± 14.15 **	252.8 ± 30.72 **
Testis	Weight	1.79 ± 0.07	1.73 ± 0.06	1.66 ± 0.10 *	1.60 ± 0.12 **
	Length	2.21 ± 0.11	2.11 ± 0.04	2.10 ± 0.06	2.08 ± 0.07 *
	Width	1.2 ± 0.06	1.16 ± 0.10	1.05 ± 0.08 **	1.05 ± 0.05 **
	Depth	1.05 ± 0.05	1 ± 0 *	0.95 ± 0.08 *	0.91 ± 0.11 *
Epididymis		1.18 ± 0.08	1.04 ± 0.07 *	0.89 ± 0.1 **	0.77 ± 0.07 **

* Values significantly different (*p* < 0.05), ** values significantly different (*p* < 0.01), control vs. Cd1, Cd2, Cd3.

**Table 2:** Effect of Cd exposure for 1, 2, and 3 months on sperm parameters.

	Control	Cd1	Cd2	Cd3
Count (×10^6^)	101.5 ± 4.42	72.53 ± 3.87 **	58.13 ± 4.25 **	35.2 ± 5.72 **
MotilityA + B (%)	81.67 ± 3.07	70.17 ± 1.16 **	54.83 ± 3.43 **	33.67 ± 2.25 **
Immotile	14.33 ± 1.86	28.0 ± 1.41 **	44.5 ± 3.08 **	65 ± 2.28 **
Viability (%)	85.67 ± 1.63	72 ± 1.41 **	59.33 ± 3.98 **	39.33 ± 2.5 **
Morphology				
Normal	87.17 ± 1.83	74.5 ± 2.07 **	61.33 ± 3.32 **	49.33 ± 5.57 **
Abnormal	12.83 ± 1.83	25.5 ± 2.07 **	38.67 ± 3.32 **	50.67 ± 5.57 **

** values significantly different (*p* < 0.01), control vs. Cd1, Cd2, Cd3.

**Table 3:** Impact of Cd exposure for 1, 2, and 3 months on plasma testosterone levels.

	Control	Cd1	Cd2	Cd3
Testosterone (ng/mL)	2.92 ± 1.21	1.91 ± 0.91	1.39 ± 0.70 *	0.80 ± 0.49 **

* Values significantly different (*p* < 0.05), ** values significantly different (*p* < 0.01), vontrol vs. Cd1, Cd2, Cd3.

**Table 4:** Cd and Zn concentrations in the testes of the control group and groups treated with Cd for 1, 2, and 3 months.

	Control	Cd1	Cd2	Cd3
Cd conc in testes (µg/g)	0.12 ± 0.10	1.36 ± 0.46 **	1.87 ± 0.49 **	2.94 ± 0.84 **
Zn conc in testes (mg/g)	0.22 ± 0.008	0.19 ± 0.007 **	0.18 ± 0.00 **	0.16 ± 0.01 **

** values significantly different (*p* < 0.01), control vs. Cd1, Cd2, Cd3.

**Table 5:** Body weight, testicular and epididymal weights, and testes dimensions in Wistar rats from experimental groups.

		Control	Cd3	Cd3recov	Cd3recov + Zn	Zn
Final body weight		307.0 ± 12.36	252.8 ± 30.72 ^a^*	299 ± 27.72	302.5 ± 17.71 ^b^*	297.3 ± 7.14
Testis	Weight	1.79 ± 0.07	1.60 ± 0.12 ^a^**	1.68 ± 0.12	1.70 ± 0.05	1.77 ± 0.04
	Length	2.21 ± 0.11	2.08 ± 0.07 ^a^*	2.10 ± 0.08	2.11 ± 0.09	2.2 ± 0
	Width	1.2 ± 0.06	1.05 ± 0.05 ^a^**	1.16 ± 0.05 ^b^**	1.16 ± 0.05 ^b^**	1.28 ± 0.07
	Depth	1.05 ± 0.05	0.91 ± 0.11 ^a^*	1.01 ± 0.04	1 ± 0 ^b^**	1.06 ± 0.05
Epididymis		1.18 ± 0.08	0.77 ± 0.07 ^a^**	0.88 ± 0.08	0.9 ± 0.07	1.12 ± 0.08

Means ± SD of six animals in each group, statistically significant differences are indicated as follows: ^a^ vs. control, ^b^ vs. Cd3, * *p* < 0, 05, ** *p* < 0, 01.

## Data Availability

The data that support the findings of this study are available from the corresponding author upon reasonable request. Funding This research received no external funding.
